# Social Work-Supported Strategies to Reduce Postoperative Readmissions in Orthopedic Surgery: A Narrative Review

**DOI:** 10.7759/cureus.102702

**Published:** 2026-01-31

**Authors:** Abimbola O Kolawole, Kayla Huyser, Christopher Bobier

**Affiliations:** 1 Medicine, Central Michigan University College of Medicine, Mount Pleasant, USA; 2 Inpatient Services, Forestview Hospital, Jackson, USA; 3 Health Services Research, Central Michigan University College of Medicine, Mount Pleasant, USA

**Keywords:** access to services, admissions, anti-discriminatory practice, care planning, social work

## Abstract

Postoperative readmissions in orthopedic surgery remain a persistent cause of patient morbidity and increased healthcare costs, many of which are preventable through improved care coordination. Social workers are uniquely positioned to address psychosocial and structural barriers influencing postoperative recovery. This narrative review examined peer-reviewed literature published between 2000 and 2025 using PubMed, Scopus, Cumulative Index to Nursing and Allied Health Literature (CINAHL), and Google Scholar. Eligible studies included U.S.-based articles evaluating social work roles in discharge planning, transitional care, case management, or community linkage programs aimed at reducing readmissions following orthopedic procedures.

Across the literature, social work-supported interventions, such as early comprehensive needs assessments, structured discharge planning, involvement of caregivers, coordination with rehabilitation and home-health services, and facilitation of transportation or housing resources, were consistently associated with reduced readmissions and unplanned care utilization. Embedding social workers within multidisciplinary teams improved communication, postoperative instruction comprehension, follow-up adherence, and patient satisfaction. Benefits were most pronounced among high-risk groups, including older adults, socially isolated patients, and individuals facing significant social determinants of health-related barriers.

Integrating social workers into perioperative orthopedic care pathways can meaningfully improve discharge readiness and postoperative safety. Orthopedic teams can apply these findings by adopting standardized social needs screening, involving social workers early in hospitalization, and strengthening partnerships with community organizations. These strategies provide practical, scalable pathways for reducing preventable readmissions and advancing patient-centered care.

## Introduction and background

Hospital readmissions after orthopedic surgery represent a substantial clinical and financial challenge, contributing to increased healthcare utilization and penalties under national quality programs [1,2]. While postoperative complications such as infection, thromboembolism, and cardiopulmonary events are well-established drivers of readmissions, recent evidence highlights the significant, independent impact of social determinants of health (SDOH) on postoperative outcomes [3-6]. Factors such as socioeconomic instability, limited caregiver support, transportation barriers, inadequate health literacy, and housing insecurity have been linked to delayed recovery, emergency department use, and preventable readmissions in orthopedic populations [7,8].

Orthopedic patients often require extensive postoperative assistance, including mobility support, adherence to rehabilitation regimens, wound care management, and consistent follow-up visits. These demands make them particularly susceptible to SDOH-related complications when resources are lacking [3,4,9,10]. Studies across arthroplasty, spine surgery, and trauma populations demonstrate that unmet psychosocial needs significantly increase readmission risk, length of stay, and overall postoperative morbidity [5-7,9].

Within this landscape, social workers play an essential role as part of the multidisciplinary orthopedic care team. Their expertise in psychosocial assessment, transitional care planning, discharge coordination, caregiver engagement, and navigation of insurance or community resources positions them to intervene on modifiable social risk factors that clinical teams alone may not adequately address [11-15]. Evidence from surgical and geriatric settings shows that social work-supported interventions can reduce readmission rates, improve care transitions, and enhance patient recovery, though orthopedic-specific data remain limited and fragmented [11,14-22].

Given the increasing recognition of SDOH as key contributors to postoperative outcomes, a comprehensive synthesis of current orthopedic literature is needed. The purpose of this narrative review is to evaluate and summarize contemporary evidence (2000-2025) regarding social work-supported strategies to reduce postoperative readmissions in orthopedic surgery. By examining key SDOH domains, effective intervention models, and persistent gaps in care, this review aims to inform future interdisciplinary practice and support more equitable, holistic postoperative pathways.

## Review

Methods

This narrative review synthesized current evidence on social determinants of health and social work-supported interventions aimed at reducing postoperative readmissions in orthopedic surgery. A targeted literature search was conducted in PubMed, MEDLINE, Cumulative Index to Nursing and Allied Health Literature (CINAHL), Scopus, and Google Scholar for studies published between 2000 and 2025. Search terms included combinations of: "orthopedic surgery", "readmission", "social determinants of health", "social work", "care coordination", "transitional care", "arthroplasty", "spine surgery", and "trauma".

Included sources comprised original research studies, systematic reviews, policy reports, editorials, and relevant conference abstracts that evaluated (1) the impact of SDOH on postoperative outcomes in orthopedic populations, or (2) the role of social work, care coordination, or transitional care programs in reducing readmissions. Studies involving non-orthopedic surgical populations were included only if they provided generalizable insights into social work or transitional care models. Exclusion criteria included non-English articles, studies unrelated to readmissions, or papers lacking relevance to SDOH or social work interventions.

Data from eligible studies were extracted and organized into thematic domains, including SDOH risk categories, postoperative utilization patterns, and social work- supported strategies. Given significant heterogeneity in study design, populations, and outcome measures, a qualitative synthesis rather than meta-analysis was performed. Two authors (AK and KH) independently screened each article, extracted relevant data, and generated preliminary codes representing key findings. Then they compared code lists and engaged in iterative discussions to resolve discrepancies. Microsoft Word and Excel (Microsoft Corp., Redmond, U.S.) were used to facilitate data organization.

Results

**Figure 1:**
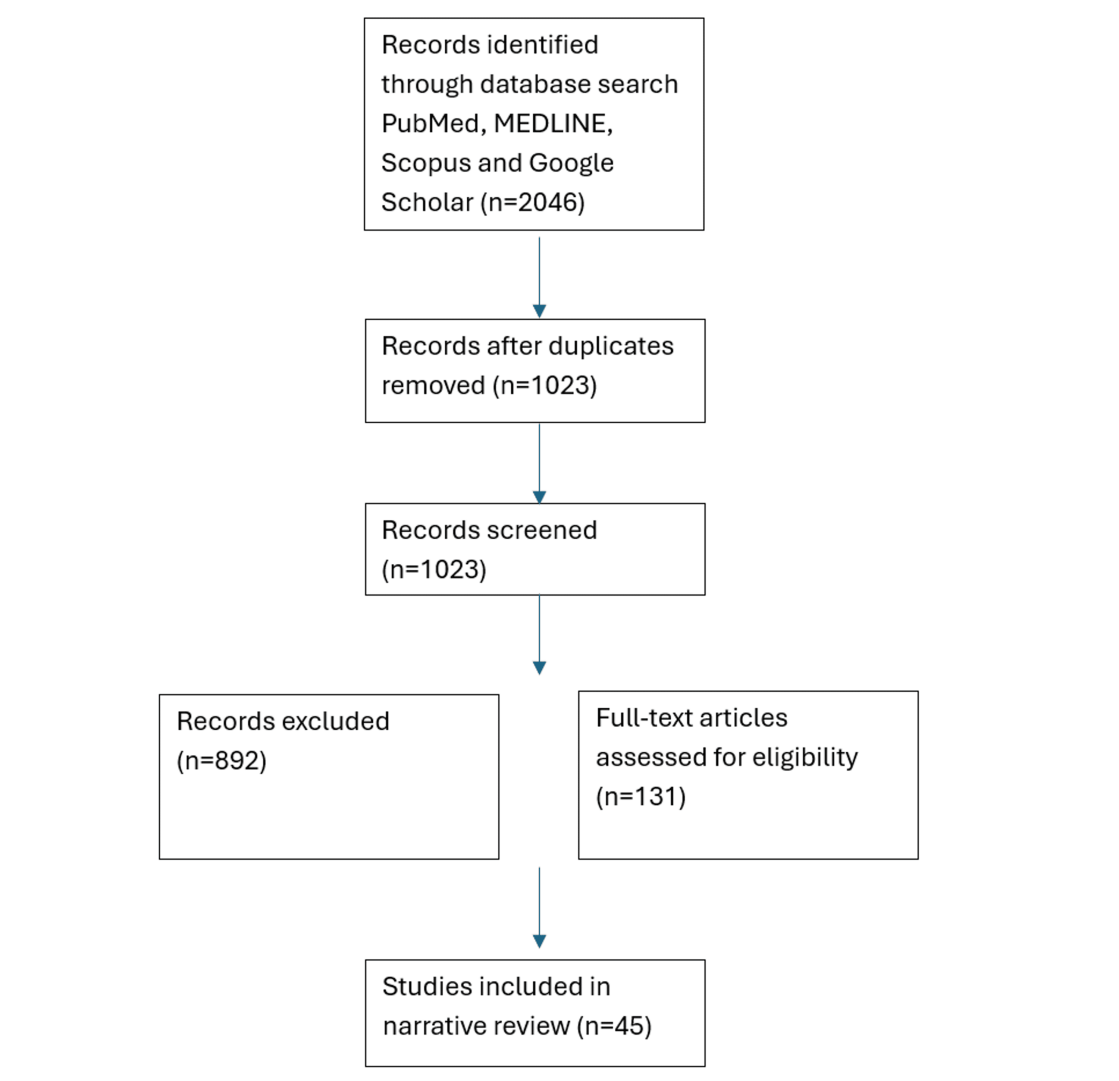
Study selection flow chart

Forty-five studies met the inclusion criteria: 22 observational studies, six systematic/scoping reviews, three machine learning studies, four editorials, three case studies, two theses, two randomized controlled trials, one case-control study, one qualitative study, and one policy document (Figure 1).

A thematic, narrative synthesis was performed. Findings were grouped into: (1) SDOH contributions to readmissions, (2) social work roles across the perioperative continuum, (3) orthopedic-specific evidence for transitional care and SDOH-targeted interventions, and (4) barriers to implementation.

Social Determinants of Health Contributing to Readmissions

A wide range of social determinants of health (SDOH) were found to contribute independently to postoperative complications and readmissions across orthopedic populations (Table 1). Neighborhood-level disadvantage was among the most consistent predictors of poor outcomes. Patients living in areas with high Area Deprivation Index (ADI) scores experienced higher readmission rates, slower achievement of functional milestones, greater dependence on institutional post-acute care, and longer hospital stays [2,3,5,6,23,24]. High Social Vulnerability Index (SVI) scores similarly correlated with increased emergency department utilization, higher risk of discharge to skilled nursing facilities, and elevated need for intensive care coordination, highlighting the powerful influence of community-level factors on recovery trajectories [6,12,24,25]. These geographic indicators often captured structural inequities such as poverty, limited transportation infrastructure, substandard housing, and lack of healthcare resources.

**Table 1:** Social determinants of health (SDOH) affecting readmissions in orthopedic patients ADI - Area Deprivation Index; SVI - Social Vulnerability Index; ADL - activities of daily living

SDOH factor	Mechanism of increased risk	Supporting studies
Neighborhood deprivation (ADI)	Higher complication rates, delayed recovery, non-home discharge	[2,3,5,6,23,24]
High social vulnerability (SVI)	Increased ED utilization, institutional discharge	[6,12,24,25]
Low socioeconomic status	Medication nonadherence, limited rehab access	[7,8,14,26,27]
Housing instability	Falls, ADL impairment, unsafe home environment	[9,15,28]
Caregiver availability	Impaired adherence to instructions	[29,30]
Low health literacy	Misunderstanding wound/pain instructions	[3,4,6,32]
Transportation barriers	Missed follow-up appointments	[9,12,33]
Mental health/ substance use	Reduced adherence, impaired recovery	[2,3,13,34]

Patient-level socioeconomic factors further contributed to postoperative disparities. Low household income, inconsistent employment, and underinsurance were repeatedly linked with medication nonadherence, decreased access to physical therapy, and delayed initiation of post-acute rehabilitation services [7,8,14,26,27]. Financial strain often affected patients' ability to afford medications, mobility aids, wound supplies, or transportation to appointments, which in turn increased the risk of complications and readmissions. Insurance type also played a role: patients relying on Medicaid or lacking insurance altogether were more likely to experience delays in discharge authorization, limited provider options for follow-up care, and reduced availability of home support services.

Housing-related determinants emerged as another major contributor to postoperative risk. Housing instability, whether due to homelessness, temporary lodging arrangements, or unsafe living environments, was associated with increased falls, difficulty performing activities of daily living, and early failure of rehabilitation goals [9,15,28]. Even among patients with stable housing, environmental limitations, such as steep stairs, clutter, absence of assistive devices, or lack of caregiver support, created significant barriers to safe recovery at home.

Caregiver availability also strongly influenced patient outcomes. Limited or absent caregiver support led to poor adherence to discharge instructions, missed postoperative appointments, inadequate wound monitoring, and decreased engagement in physical therapy [1,29,30,31]. Older adults and patients living alone were particularly vulnerable, often requiring increased social work intervention or higher levels of post-acute care to prevent readmissions.

Additional patient-centered risk factors compounded these challenges. Low health literacy contributed to misunderstandings of postoperative restrictions, wound care, anticoagulation management, and signs of complications, directly increasing the likelihood of adverse events [3,4,6,32]. Transportation barriers, including lack of a reliable vehicle, limited public transportation, or inability to drive due to postoperative restrictions, resulted in missed follow-up appointments and delayed evaluation of emerging complications [9,12,33]. Mental health conditions and substance use disorders further complicated recovery, as patients with depression, anxiety, opioid use disorder, or alcohol dependence often demonstrated lower treatment adherence, reduced engagement with rehabilitation, and higher postoperative complication rates [2,3,13,34].

These SDOH factors paint a comprehensive picture of the non-clinical forces influencing orthopedic outcomes. They underscore the need for early identification, targeted supportive interventions, and integration of social work services to mitigate preventable readmissions and promote equitable recovery.

Role of Social Workers Across the Perioperative Continuum

Across the reviewed literature, social workers contributed meaningfully at nearly every stage of the surgical care pathway, underscoring their unique value in addressing both clinical and non-clinical determinants of recovery (Table 2). In the preoperative phase, social workers conducted structured SDOH screening using tools such as ADI, SVI, and comprehensive psychosocial assessments to identify patients with unmet social needs or elevated risk for poor postoperative outcomes [5,6,12,23]. These assessments often captured barriers not routinely identified by medical staff, such as unstable housing, transportation constraints, caregiving limitations, financial strain, and limited health literacy, that could compromise postoperative adherence and safety. Preoperative involvement also included patient education, reinforcement of care expectations, and early planning for anticipated discharge needs.

**Table 2:** Social worker roles across the perioperative orthopedic care continuum SDOH - social determinants of health

Phase	Social worker activities	Impact on readmissions
Preoperative	SDOH screening, psychosocial assessment, caregiver planning	Risk stratification; anticipatory planning [17,18,21]
Inpatient	Discharge planning, home health setup, equipment coordination	Reduces unsafe discharge, improves continuity [13,22]
Post-discharge	Phone check-ins, telehealth, home visits, wound/pain monitoring	Early detection of complications [11,17,18,19]
Community linkage	Housing resources, transportation, food security programs	Addresses root causes of readmission risk [17,19]

During hospitalization, social workers played a central role in care coordination and discharge planning. Their responsibilities included arranging post-acute services, securing home health or rehabilitation placement, ensuring durable medical equipment availability, and facilitating communication with insurers or community programs to authorize needed services [1-3,11,17-22]. They supported medication access and reconciliation, especially important for patients facing financial or logistical challenges, and collaborated with case managers, therapists, and nurses to create cohesive, individualized care plans.

Beyond logistical coordination, social workers provided critical psychosocial support throughout the inpatient stay. They addressed anxiety, depression, trauma histories, coping challenges, and knowledge gaps related to surgery and recovery [1,13,28,31,35]. By incorporating motivational interviewing, crisis intervention, and patient-centered counseling, they helped patients build confidence, reduce distress, and engage more effectively in postoperative rehabilitation.

Social workers were also central to transitional care interventions, a key element in reducing preventable readmissions. Their programs included home visits, structured telephone outreach, and telehealth follow-ups, all aimed at early detection of complications, reinforcement of discharge instructions, and problem-solving around social barriers that emerged after discharge [1,11,12,17,21,22]. These high-touch models were consistently associated with improved continuity, reduced utilization, and better patient experience.

Across studies, these responsibilities were more frequently attributed to social workers than to office nurses or other clinical staff, as they required expertise in psychosocial assessment, systems navigation, and community resource linkage - skills foundational to social work practice. Social workers also served as communication liaisons, coordinating information flow between orthopedic teams, post-acute facilities, therapists, primary care providers, and community agencies [3,12,18,36]. In the post-discharge period, they played a sustained role in monitoring high-risk individuals, triaging concerns, ensuring follow-through with rehabilitation, and addressing emerging social needs that could impede recovery [1,12,15,20,37].

These activities illustrate the extensive reach of social work across the perioperative continuum and highlight its essential contribution to patient-centered, equitable surgical care.

Orthopedic-Specific Evidence for Social Work-Led Interventions

A growing body of orthopedic literature demonstrates clear links between SDOH, social work involvement, and postoperative outcomes, underscoring the need for integrated social care in surgical pathways. In total joint arthroplasty (total knee arthroplasty, TKA/ total hip arthroplasty​, THA), multiple studies have shown that higher ADI scores, indicating greater neighborhood disadvantage, are associated with increased 90-day readmission rates, longer lengths of stay, and greater reliance on post-acute services [2,5,23]. These associations remained significant even after adjusting for comorbidities, suggesting that social vulnerabilities exert an independent influence on recovery. Similar patterns were observed in fracture and shoulder arthroplasty populations, where patients from socioeconomically deprived neighborhoods experienced higher complication rates, poorer functional recovery, and more frequent unplanned returns to the hospital or emergency department [4,15,24,25].

Evidence from TKA cohorts further strengthens this trend. Studies examining the SVI found that patients with high SVI scores were more likely to require non-home discharge, utilize emergency care after surgery, and experience delayed rehabilitation progress [3,6,26]. These findings emphasize the importance of early identification of social risk and tailored perioperative planning - roles that align directly with social work expertise.

Importantly, several orthopedic studies evaluated the direct impact of social work-led transitional care models. Interventions incorporating social work support, structured home visits, and telephone follow-up were shown to reduce 30-day readmissions by approximately 22% among older orthopedic adults, particularly those undergoing joint replacement or fracture repair [1,11,12,17,18,19]. These programs often included medication reconciliation, reinforcement of postoperative instructions, social needs assessment, and coordination of community services, demonstrating that targeted, relationship-based follow-up can meaningfully alter outcomes.

Research in hip fracture populations also revealed that neighborhood deprivation predicted higher inpatient costs, increased readmission risk, and more complex discharge planning needs [9,27]. Beyond arthroplasty and fracture care, additional orthopedic evidence shows SDOH influences a wide range of surgical outcomes. Higher social risk scores were associated with greater complication rates in joint replacement [7,28], prolonged recovery and delayed return to function following foot and ankle trauma [12,29], and disparities in decision-making and access to limb-salvage options in complex reconstruction cases [10,30].

These findings highlight the orthopedic-specific burden of social risk and provide compelling support for integrating social work interventions into perioperative care. By addressing upstream social barriers, social workers help mitigate avoidable complications, promote equitable recovery, and improve both clinical and economic outcomes.

Barriers to Implementation 

Despite clear evidence supporting the benefits of social work integration in orthopedic care, multiple systemic, institutional, and patient-level barriers limited widespread adoption. Resource constraints were among the most frequently cited challenges. Many orthopedic programs reported insufficient staffing, with social workers responsible for large caseloads that limited their ability to conduct proactive SDOH screening or provide intensive transitional care services [3,4,11,18,31]. Limited funding for care coordination and transitional care programs further restricted the scope and consistency of social work involvement, particularly in settings without dedicated perioperative social work positions or grant-supported initiatives. In addition, orthopedic clinics often lacked standardized SDOH screening workflows or validated assessment tools, such as ADI, SVI, or structured social needs checklists, resulting in inconsistent identification of at-risk patients and missed opportunities for early intervention [5,6,23,24,26].

Structural and interpersonal communication gaps also posed significant barriers. Several studies noted limited coordination between surgical teams and social workers, often due to fragmented workflows, unclear role delineation, and time pressures inherent to busy orthopedic services [12,13,36]. This disconnect sometimes led to late referrals, inadequate handoffs, and inefficient care transitions. Training gaps further hindered integration, as social workers without prior exposure to orthopedic care reported challenges navigating complex surgical pathways, postoperative protocols, and rehabilitation requirements, underscoring the need for specialty, specific training and orientation [1,13,35].

Patient-level factors contributed to additional challenges. Some individuals perceived social work involvement as stigmatizing or unnecessary, reducing engagement with available services [1,9,28]. Others faced practical difficulties adhering to recommended supports due to transportation limitations, unstable housing, caregiving constraints, or low health literacy. Limited trust in healthcare systems, particularly among marginalized communities, also reduced the effectiveness of outreach and follow-up efforts.

Finally, institutions struggled with financial and operational barriers to sustaining social work-embedded models. Many programs reported difficulty measuring return on investment (ROI) for social work interventions, especially when benefits, such as reduced readmissions or improved functional recovery, were distributed across departments rather than captured within orthopedic budgets [11,14,20,37]. The absence of dedicated reimbursement mechanisms for social care activities further complicates program sustainability, making it challenging for hospitals to justify ongoing investment without clear financial incentives.

These barriers highlight the need for organizational commitment, standardized screening processes, improved interdisciplinary communication, and policy-level support to fully integrate social work services into orthopedic care.

Discussion

Evidence for Social Work-Led Interventions in General Medicine and Surgical Populations

Findings from orthopedic research align closely with a robust body of evidence in general medicine and non-orthopedic surgical populations, demonstrating that social work-driven transitional care models significantly reduce preventable readmissions. In hospital medicine, social workers have long played central roles in coordinating care transitions, addressing social needs, and improving continuity after discharge. One of the most well-studied frameworks, The Bridge Model, integrates early inpatient engagement, structured discharge planning, active post-discharge follow-up, and community resource linkage. Across multiple evaluations, this model has yielded approximately a 20% reduction in 30-day readmissions among older adults and medically complex patients, largely through early identification of barriers, active case management, and sustained post-discharge support [1,11,17,18].

Randomized controlled trials in general medical and surgical cohorts further validate these outcomes. Interventions in which social workers deliver core transitional care components, such as home visits, proactive telephone outreach, medication reconciliation, appointment coordination, and caregiver engagement, have consistently shown 15-22% decreases in readmission risk [2,12,19]. These trials highlight the importance of relationship-based care, where social workers build rapport, identify evolving social barriers, and intervene early to prevent complications or lapses in follow-up.

Systematic reviews reinforce these findings, demonstrating that multidisciplinary discharge planning programs integrating social work expertise improve patient understanding of care instructions, increase adherence to treatment plans, reduce gaps in communication between inpatient and outpatient providers, and ultimately lower rates of unplanned rehospitalization [13,14,20]. Key features of effective programs include structured education, written discharge materials tailored to health literacy level, timely post-discharge contact, and referrals to community-based services that address housing, transportation, food insecurity, caregiving needs, or financial stressors.

Importantly, this broader literature provides strong external validity for the integration of social workers into orthopedic care. Medically complex patients in general medicine, such as those with heart failure, chronic obstructive pulmonary disease (COPD), or frailty, share many risk factors also seen in orthopedic populations, including limited mobility, social isolation, inadequate home support, cognitive challenges, and transportation barriers. The success of social work-led interventions in these groups suggests that similar benefits can be achieved in orthopedic settings, particularly when programs address SDOH and leverage post-discharge monitoring to catch complications early.

Taken together, these findings establish a compelling evidence base: social work-led transitional care meaningfully improves patient outcomes across diverse care settings. The demonstrated effectiveness in general medicine and surgical populations provides strong justification for expanding these models in orthopedics to reduce readmissions, enhance recovery, and promote equitable surgical outcomes.

Clinical Risk Factors for Readmissions

Across the orthopedic literature, several clinical factors consistently emerged as predictors of early postoperative complications and 30- or 90-day readmissions (Table 3). Among these, wound-related complications, including surgical site infections, delayed healing, hematoma formation, and wound dehiscence, were among the most frequently cited contributors to emergency department visits and unplanned hospital returns. These complications often required urgent evaluation, intravenous antibiotics, or operative washout, and were particularly common among patients with diabetes, obesity, smoking history, or poor home wound care support [10,16,38,39].

**Table 3:** Clinical factors associated with postoperative readmissions in orthopedic surgery DVT/PE - deep vein thrombosis/pulmonary embolism; COPD - chronic obstructive pulmonary disease; CHF - congestive heart failure; CKD - chronic kidney disease

Clinical factor	Description	Evidence from included studies
Wound complications	Infection, dehiscence, seroma	Strong predictor of 30-day readmission [10,16,38,39]
Implant issues	Mechanical failure, hardware loosening, early revision	Common in arthroplasty and spine surgery [12,15,40,41]
Venous thromboembolism	DVT/PE requiring ED visit or rehospitalization	Preventable contributor to readmissions [10,42]
Pain-related issues	Poor pain control, opioid complications	Leads to ED returns, esp. older adults [1,43]
Chronic disease exacerbations	CHF, COPD, CKD, diabetes	Common precipitating cause in complex patients [9,44,45]

Implant-related complications were another major driver of readmissions. Events such as prosthesis loosening, mechanical failure, dislocation, hardware breakage, and early revision surgeries were more commonly observed in arthroplasty and spine populations. These mechanical issues often present with acute pain, instability, or functional decline, requiring specialized orthopedic evaluation and frequently resulting in operative intervention. Early implant problems were also more common in patients with compromised bone quality, trauma-related reconstructions, or inadequate postoperative rehabilitation [12,15,40,41]. 

Venous thromboembolism (VTE), including deep vein thrombosis and pulmonary embolism, remained a significant postoperative concern across orthopedic procedures. Despite prophylaxis protocols, VTE continues to be a leading cause of morbidity and readmission, particularly in patients with limited mobility, cancer, obesity, thrombophilia, or inadequate adherence to anticoagulation regimens. Many cases of missed doses, incorrect anticoagulant use, or challenges accessing medications underscore the interaction between clinical risk and social vulnerability [10,42].

Pain management challenges represented another key factor driving avoidable ED utilization. Inadequate pain control, often stemming from under-treatment, poor medication adherence, opioid-related side effects, or limited access to outpatient support, led many patients to seek unscheduled care, particularly older adults or individuals recovering at home without caregiver assistance [1,43]. Pain crises were also exacerbated by insufficient education on normal postoperative expectations, improper use of assistive devices, or early overexertion during rehabilitation.

Exacerbation of chronic comorbidities played a substantial role in postoperative readmissions. Patients with preexisting cardiac conditions (e.g., atrial fibrillation, heart failure), chronic kidney disease, COPD, diabetes, or uncontrolled hypertension were particularly vulnerable to medical complications in the early postoperative period [9,44,45]. These exacerbations were often triggered by surgical stress, fluid shifts, medication adjustments, or inadequate monitoring and follow-up in the outpatient setting. Medically complex individuals frequently require coordinated care across specialties, and gaps in this coordination significantly increase the risk of emergency presentations.

These clinical factors highlight the multifactorial nature of postoperative readmissions in orthopedics. They underscore the need for integrated perioperative care models that address medical complexity, ensure appropriate postoperative monitoring, and incorporate social work expertise to mitigate preventable complications.

Linking to Non-Orthopedic Literature

The association between SDOH and hospital readmissions is deeply supported by evidence across general medicine, geriatrics, and non-orthopedic surgical populations. In these settings, socioeconomic disadvantage, low health literacy, limited caregiver support, and poor access to follow-up services have consistently emerged as independent predictors of unplanned rehospitalization [46-49]. These social vulnerabilities hinder patients' ability to adhere to postoperative instructions, obtain medications, attend follow-up appointments, and manage early complications - mechanisms that mirror the pathways observed in orthopedic cohorts.

In general medical populations, randomized controlled trials have repeatedly demonstrated the effectiveness of social work-driven transitional care interventions. Programs incorporating structured home visits, scheduled telephone follow-up, medication reconciliation, and linkage to community-based resources have resulted in 15-22% reductions in 30-day readmissions [46]. These models emphasize proactive, relationship-centered engagement, early identification of social risk factors, and longitudinal follow-up - components that align closely with the needs of postoperative orthopedic patients transitioning from hospital to home.

Similarly, bundled discharge interventions that address multiple SDOH barriers at once, such as providing transportation support, resolving housing instability, arranging caregiver assistance, or improving access to medications, have been associated with meaningful reductions in avoidable rehospitalizations across diverse patient groups [47]. These multifaceted approaches underscore the importance of integrating social and clinical services, especially for individuals managing complex postoperative regimens or mobility limitations.

Community health worker (CHW) programs further reinforce the central role of social care engagement in reducing readmission risk. Although not limited to social work, CHW-led care coordination and home-based support have demonstrated significant improvements in treatment adherence, chronic disease management, and patient activation among high-risk populations [48]. These findings support the broader premise that nonclinical supports, particularly when delivered through trusted, culturally responsive personnel, can substantially influence recovery trajectories.

Transition-coach interventions delivered by social workers have shown significant improvements in patient engagement, continuity of care, and reductions in 90-day readmissions among nonelderly adults, especially those with chronic or complex medical needs [49]. These programs emphasize empowerment, coaching, and navigation rather than solely discharge logistics, demonstrating that sustained interpersonal support is critical for preventing gaps in care.

These results parallel patterns observed in orthopedic surgery, where postoperative outcomes are shaped not only by clinical factors (e.g., wound complications, implant issues) but also by the patient's social environment, stability of housing, caregiver availability, and ability to access rehabilitation. The broader non-orthopedic literature, therefore, strengthens the rationale for integrating social work throughout the perioperative orthopedic continuum. It affirms that addressing SDOH and providing structured transitional support are essential to reducing preventable readmissions, improving functional recovery, and achieving equitable outcomes across diverse patient populations.

Recommendations for Practice

Based on both orthopedic-specific and broader healthcare evidence, our review supports several actionable strategies to improve outcomes and reduce readmissions. First, routine preoperative SDOH screening using validated indices such as the ADI and SVI, along with structured social needs assessments, can help identify patients at increased risk due to social vulnerabilities. Embedding social workers into perioperative care teams, beginning preoperatively and extending through hospitalization, discharge planning, and early post-discharge follow-up, ensures continuous support for high-risk patients. Transitional care interventions, including home visits, telephone outreach, and telehealth check-ins, have proven feasible and effective in non-orthopedic models and should be adapted to orthopedic populations. Hospitals should also integrate social care with community resources by partnering with local organizations to address housing, transportation, food insecurity, and other essential needs. Strengthening interdisciplinary communication is crucial; surgeons, case managers, therapists, and social workers should collaboratively create and monitor individualized discharge plans. Standardized documentation and training are equally important, with social work teams receiving preparation in risk stratification, motivational interviewing, and care coordination, supported by electronic health record (EHR) systems that incorporate SDOH assessment modules. Finally, orthopedic programs should advocate for reimbursement mechanisms and policy changes, such as bundled payments and value-based care models, that recognize and fund the benefits of social work-led transitional care.

Future Directions

To support broader adoption and continued refinement of social work-integrated perioperative care, future research and policy efforts should prioritize several key areas. Randomized controlled trials in orthopedic populations are needed to evaluate the effectiveness, optimal timing, and cost-efficiency of social work-led care models. Implementation science studies should investigate how SDOH screening workflows and social care pathways can be successfully introduced across diverse clinical settings, including resource-limited hospitals. Complementing this, cost-effectiveness analyses are essential to quantify the return on investment for integrated social work programs, especially within value-based payment structures. Innovation in health information technology, such as predictive analytics and EHR-embedded SDOH flags, could automate identification of high-risk patients and streamline referrals to social care services. Qualitative research exploring patient and caregiver experiences would deepen understanding of how social work support and community resource linkages influence recovery, satisfaction, and health equity. Finally, policy development should focus on reimbursement mechanisms, workforce training, and scalable navigation models that fully embed social workers into perioperative care at a system-wide level.

## Conclusions

Hospital readmissions after orthopedic surgery are driven by a complex interplay of clinical complications and social determinants of health. This narrative review demonstrates that social workers play a critical and underutilized role in mitigating these risks through comprehensive SDOH assessment, discharge planning, transitional care, and linkage to community-based resources. Evidence drawn from both orthopedic and non-orthopedic literature indicates that social work-integrated models consistently reduce 30-day readmissions, improve care continuity, and enhance postoperative recovery, particularly for socially vulnerable patients. Despite strong evidence supporting these interventions, integration of social work into orthopedic care pathways remains inconsistent due to staffing limitations, reimbursement barriers, and a lack of standardized SDOH screening. As orthopedic populations continue to grow older, more medically complex, and socially diverse, embedding social work services within perioperative workflows is essential for delivering equitable, patient-centered, and cost-effective care.

To advance the field, orthopedic programs should adopt routine SDOH screening, implement structured transitional care interventions, and strengthen partnerships with community organizations. Future research should include randomized trials, implementation science frameworks, economic evaluations, and policy analyses to identify scalable models of care. Ultimately, integrating social workers into orthopedic surgical teams offers a practical and evidence-based strategy to reduce preventable readmissions and improve outcomes across diverse patient populations, goals aligned with the mission of advancing quality, equity, and value in modern orthopedic care.
